# Supplementary material for: Treatment of atherosclerosis by macrophage-biomimetic nanoparticles via targeted pharmacotherapy and sequestration of proinflammatory cytokines
Source: Nat Commun. 2020 May 26;11:2622. doi: 10.1038/s41467-020-16439-7 (PMC7251120; doi:10.1038/s41467-020-16439-7)
Supplement: Supplementary file 1 — Supplementary Information [file 41467_2020_16439_MOESM1_ESM.pdf]

Supplementary Information

Treatment of atherosclerosis by macrophage-biomimetic nanoparticles via targeted pharmacotherapy and sequestration of proinflammatory cytokines

Gao et al.

List of supplementary figures

Supplementary Figure 1. Synthetic process and ROS responsiveness of Oxi-COS.

Supplementary Figure 2. ^{1}H NMR spectra of compound **1**, compound **2**, COS and Oxi-COS.

Supplementary Figure 3. Characterization of MM-NPs.

Supplementary Figure 4. Attenuation effects of MM-AT-NPs on oxLDL (or LPS) treated RAW264.7 cells.

Supplementary Figure 5. The phenotypic changes of RAW264.7 cells.

Supplementary Figure 6. Optical microscope images of oxLDL induced foam cells.

Supplementary Figure 7. Cellular uptake of Cy5-NPs by LPS induced foam cell. Scale bar: 50 μm.

Supplementary Figure 8. In vivo safety evaluation of MM-NPs.

Supplementary Figure 9. Safety evaluation of RAW264.7 cells in ApoE$^{-/-}$ mouse (intravenously administered with macrophage).

Supplementary Figure 10. Characterization of AT-NPs internalized macrophages.

Supplementary Figure 11. *In vivo* pharmacokinetics of Cy-NPs, MM-Cy7.5-NPs and Cy7.5-NPs/MAs in mouse model.

Supplementary Figure 12. Representative ORO stained with sequential 10 cryosections at 100 μm intervals from the aorta roots, and average plaque area in each section determined by Image Pro.

Supplementary Figure 13. Representative photographs and quantitative analysis of aorta root sections stained by CD31 antibody and KI67 antibody.

Supplementary Figure 14. Anti-atherosclerotic mechanisms by MM-AT-NPs.

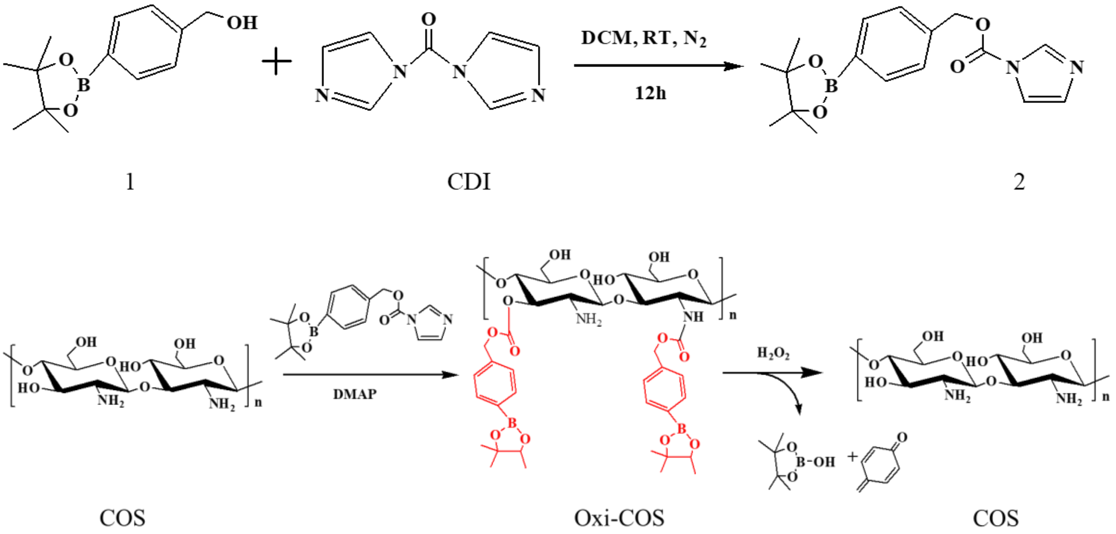

Supplementary Figure 1. Synthetic procedure and ROS responsiveness of Oxi-COS.

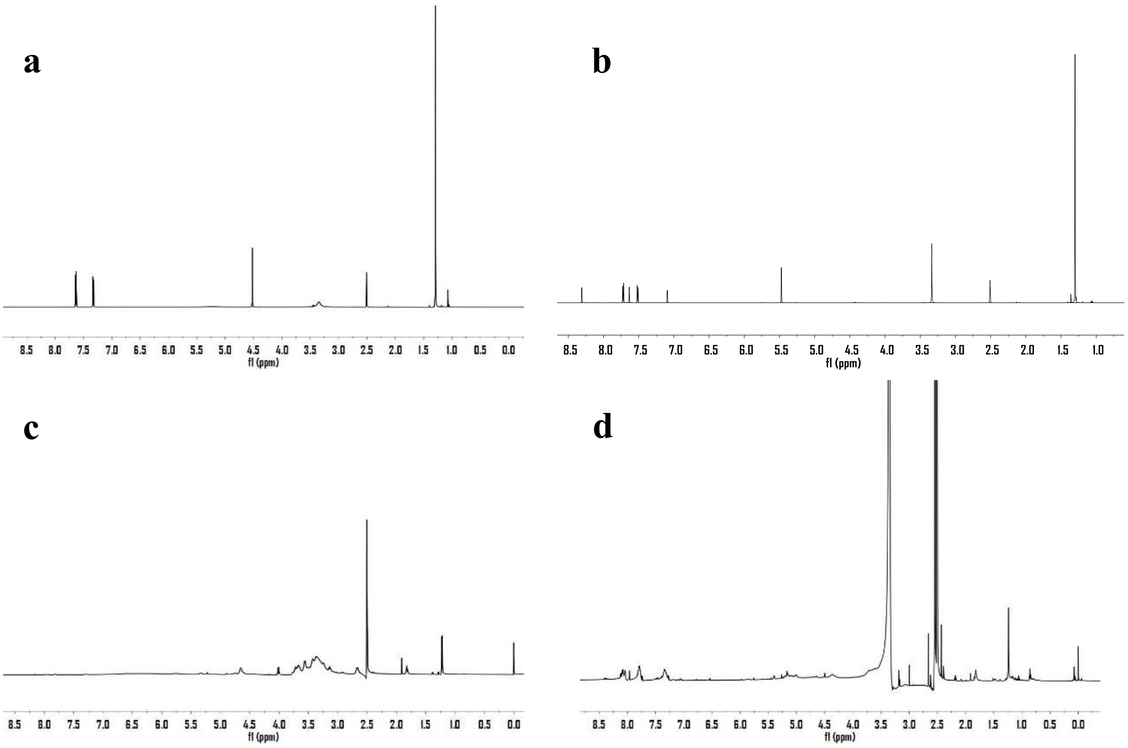

Supplementary Figure 2. ^{1}H NMR analysis. ^{1}H NMR spectra of compound **1** (**a**), compound **2** (**b**), COS (**c**) and Oxi-COS (**d**) in DMSO-d6.

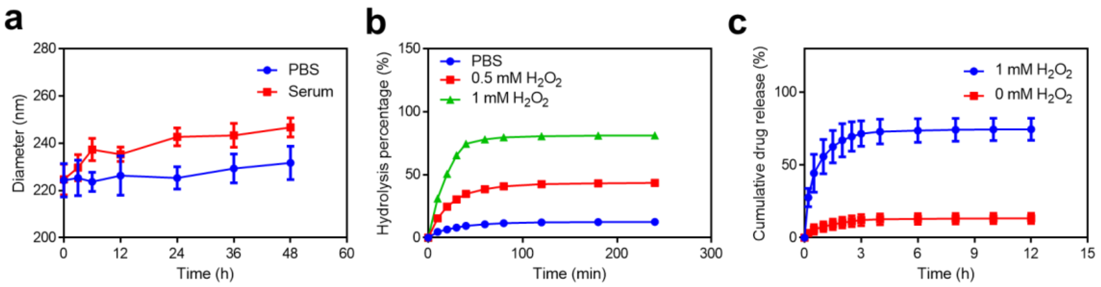

Supplementary Figure 3. Characterization of MM-NPs. a, Diameter changes of MM-NPs placed in PBS and serum for 48 h, respectively. The date was obtained by three independent experiments (n=3) and presented as mean ± s.d. **b**, MM-NPs were placed in H_2O_2 (0.5 mM and 1 mM) and the hydrolysis rate was detected by UV spectrum at predetermined time intervals. **c**, In vitro drug release profile of MM-AT-NPs in 1 mM of H_2O_2. The experiments were repeated for three times (*n* = 3) and data was presented as mean ± s.d. Source data are provided as a Source Data file.

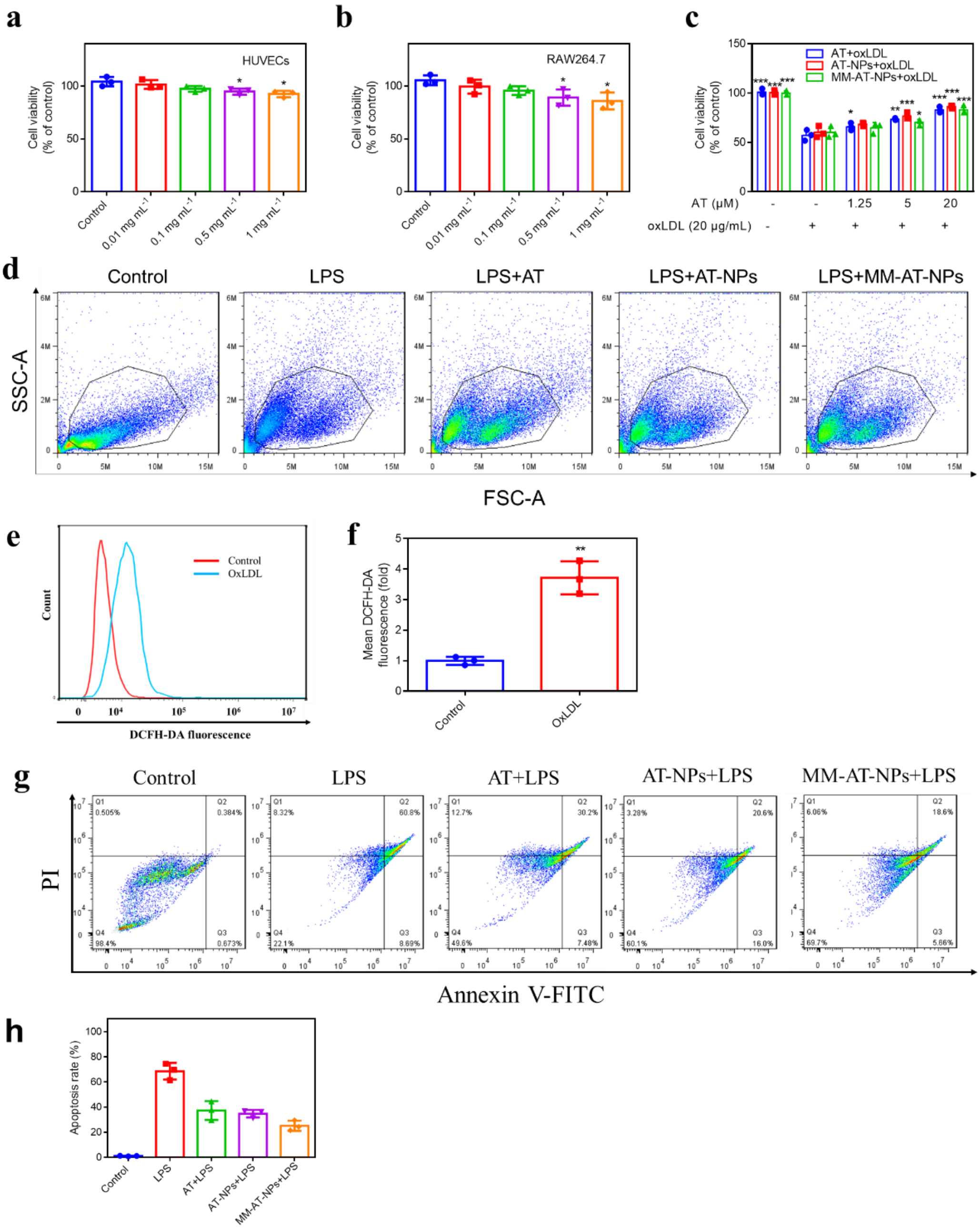

Supplementary Figure 4. Attenuation effects of MM-AT-NPs on oxLDL (or LPS) treated RAW264.7 cells. **a**,**b**, Safety evaluation of blank NPs in HUVECs and RAW264.7 cells. **c**, HUVECs were co-treated with oxLDL (20 μg mL^{-1}) and three AT formulations (AT, AT-NPs and MM-AT-NPs) at different AT concentrations (1.25μM, 5μM and 20μM). **d**, Gating strategy used for ROS producing experiment of RAW264.7 cells. **e**, Intracellular ROS levels in HUVECs treated with oxLDL (20 μg mL^{-1}). **f**, Quantitative analysis of ROS levels by flow cytometry. **g**,**h**, Apoptosis rates of RAW264.7 cells, induced by LPS (400 ng mL^{-1}), w or w/o each of three AT formulations (AT, AT-NPs and MM-AT-NPs), respectively at 0.4 mM AT for 24 h, analyzed by flow cytometry. The experiments were repeated for three times ($n = 3$) and data were presented as mean ± s.d. Statistical analysis for cell viability of HUVECs co-treated with oxLDL and AT formulations was performed using Two-Way ANOVA. Analysis for safety evaluation, and DCFH-DA fluorescence were conducted using One-Way ANOVA. *P≤0.05, **P≤0.01, and ***P≤0.001. Source data are provided as a Source Data file.

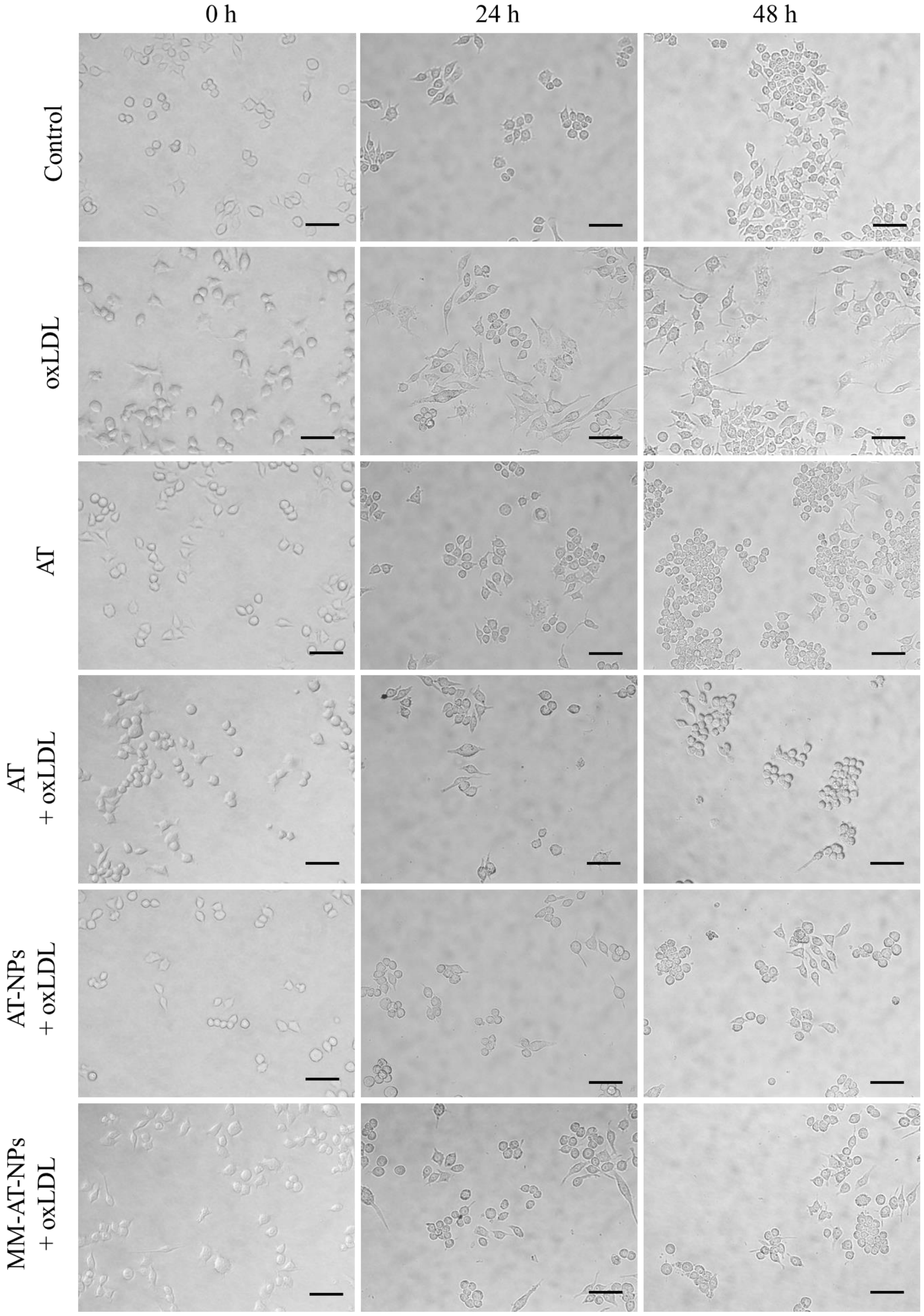

Supplementary Figure 5. The phenotype changes of RAW264.7 cells. Various AT formulations (AT, AT-NPs and MM-AT-NPs) inhibited the oxLDL-induced immature dendritic cells-like morphologic changes in RAW264.7 cells. RAW264.7 cells were treated with oxLDL (20 μg/ml) in the absence or in the presence of various AT formulations (20 μM) for 48 h. The experiments were conducted independently for three times. Scale bar: 50 μm.

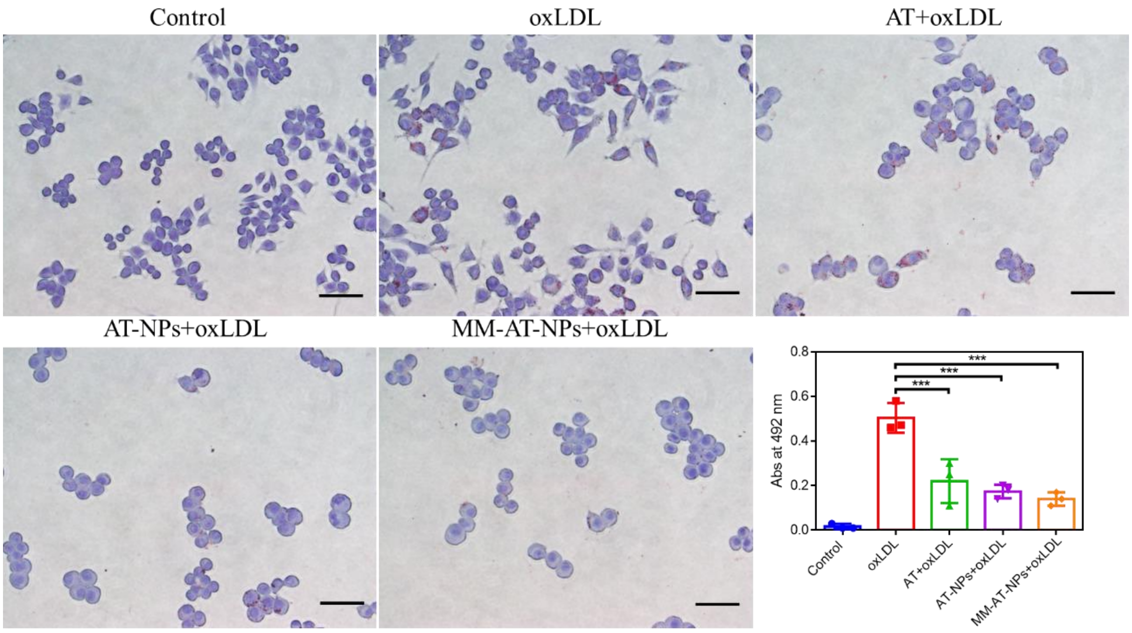

Supplementary Figure 6. Optical microscope images of oxLDL induced foam cells. RAW264.7 cells were co-treated with oxLDL (20 μg/ml) in the absence or in the presence of various AT formulations (20 μM) for 48 h. Then, the cells were stained with ORO for microscopic observation, and the intracellular ORO was dissolved with isopropyl alcohol for quantification. Scale bar: 50 μm. The experiments were conducted independently for three times and data were presented as mean ± s.d (*n* = 3). Statistical analysis was performed using One-Way ANOVA. *** P≤0.001. Source data are provided as a Source Data file.

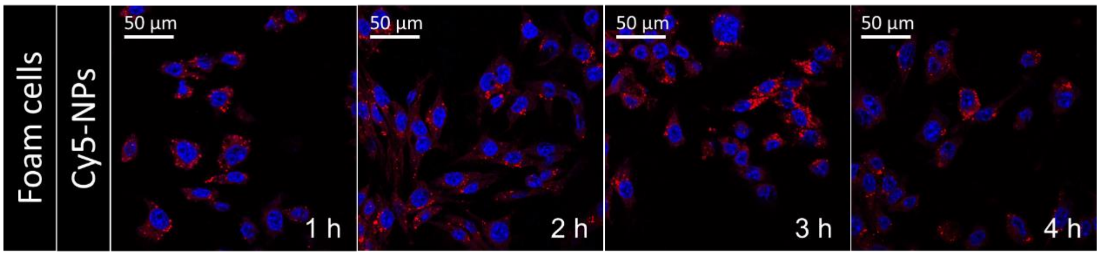

Supplementary Figure 7. Cellular uptake of Cy5-NPs by LPS induced foam cell. The experiments were conducted independently for three times. Scale bar: 50 µm.

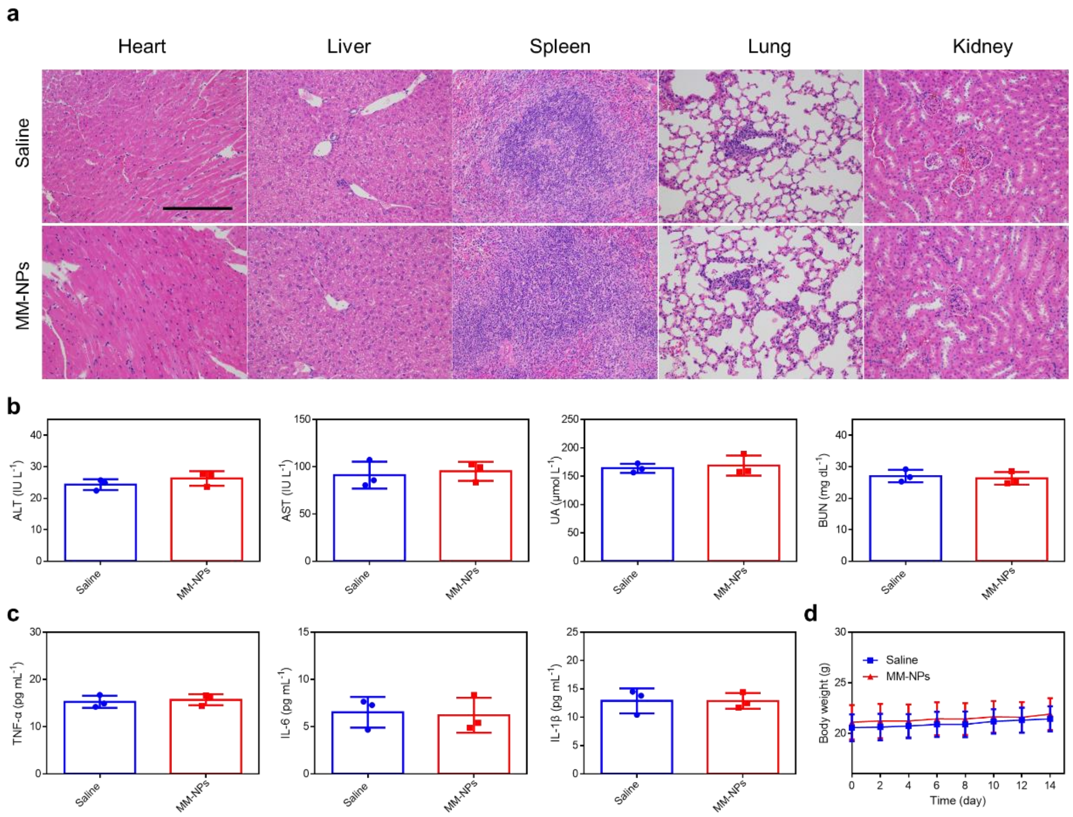

Supplementary Figure 8. *In vivo* safety evaluation of MM-NPs. a, The C57BL/6 mice were i.v. injected with saline and MM-NPs for half a month with a high dose of 100 mg kg-1 MM-NPs every four days, and heart, liver, spleen, lung and kidney were collected for histological studies. Scale bar: 200 μm. **b**, The liver function biomarkers (ALT and AST) and kidney function biomarkers (BUN and UA) in the serum of mice treated with MM-NPs were measured. **c**, The inflammatory cytokine levels (TNF-α, IL-6 and IL-1β) in the serum of mice treated with MM-NPs were measured by Elisa assay kits. **d**, The changes of bodyweight in mice treated with MM-NPs were recorded. All experiments were repeated for three times (n = 3) and data was presented as mean ± s.d.

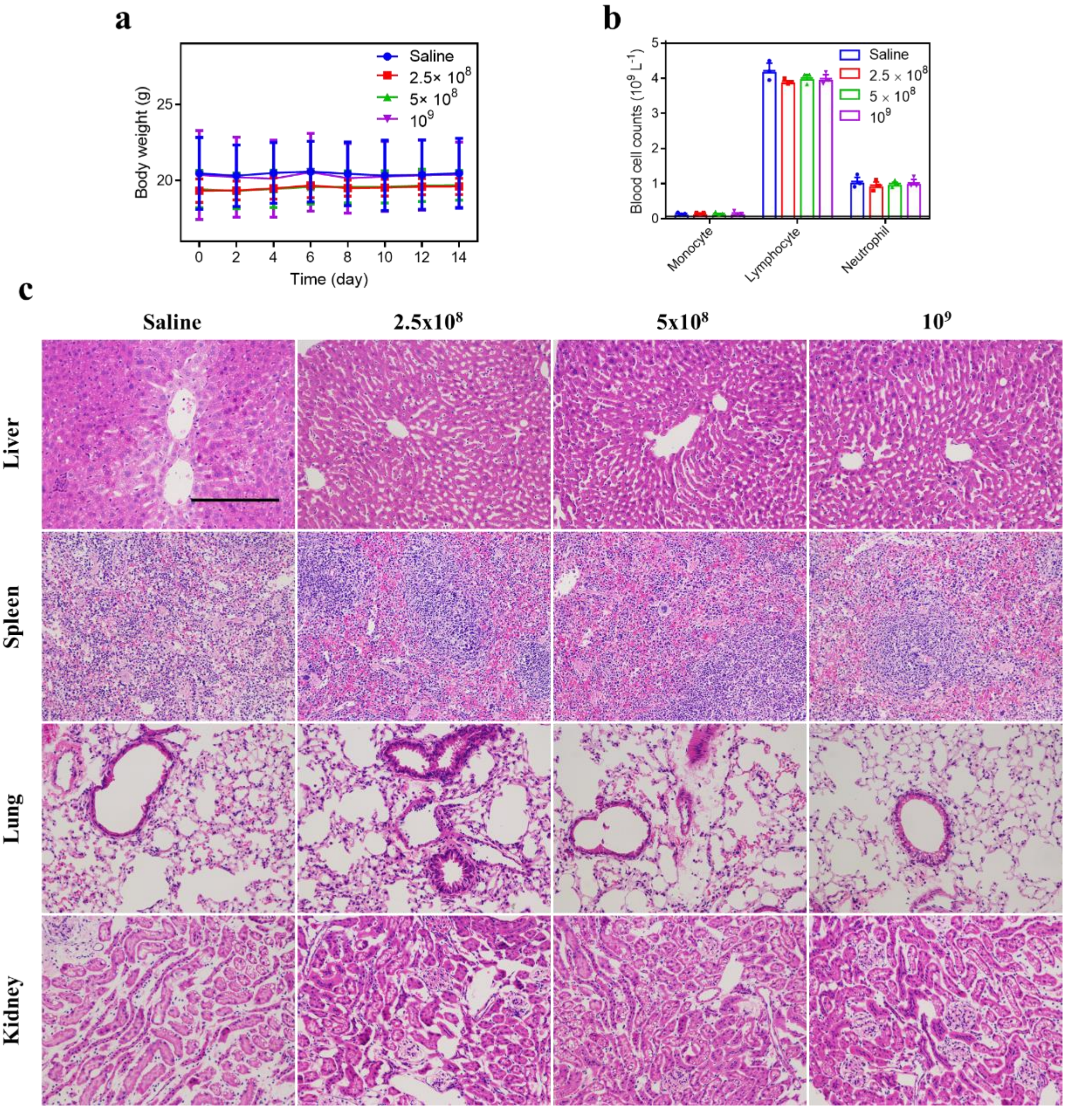

4.7

Supplementary Figure 9. Safety evaluation of ApoE$^{-/-}$ mouse intravenously administered with RAW264.7 cells. a, Changes of body weight. **b**, Blood cell counts of immune-associated cells including monocyte, lymphocyte and neutrophil. **c**, Histological studies of the liver, spleen, lungs and kidneys. Scale bar: 200 μm. The experiments were repeated for three times ($n = 3$) and data were presented as mean ± s.d. Statistical analysis for blood cell counts was performed using Two-Way ANOVA and no statistical significance was observed in monocyte, lymphocyte and neutrophil between groups.. Source data are provided as a Source Data file.

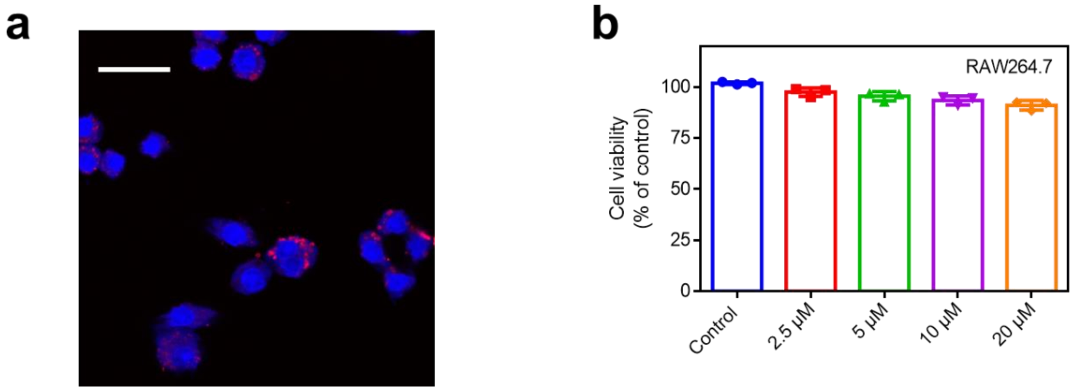

Supplementary Figure 10. Characterization of AT-NPs internalized macrophages. **a**, Representative image of Cy7.5-NPs/MAs determined by laser scanning confocal microscope. The experiments were conducted independently for three times. Scale bar: 50 μm. **b**, RAW264.7 cells were incubated with AT-NPs at different concentrations of AT (2.5 μM, 5 μM, 10 μM and 20 μM) for 24 h. The experiments were repeated for three times (n = 3) and data was presented as mean ± s.d. Source data are provided as a Source Data file.

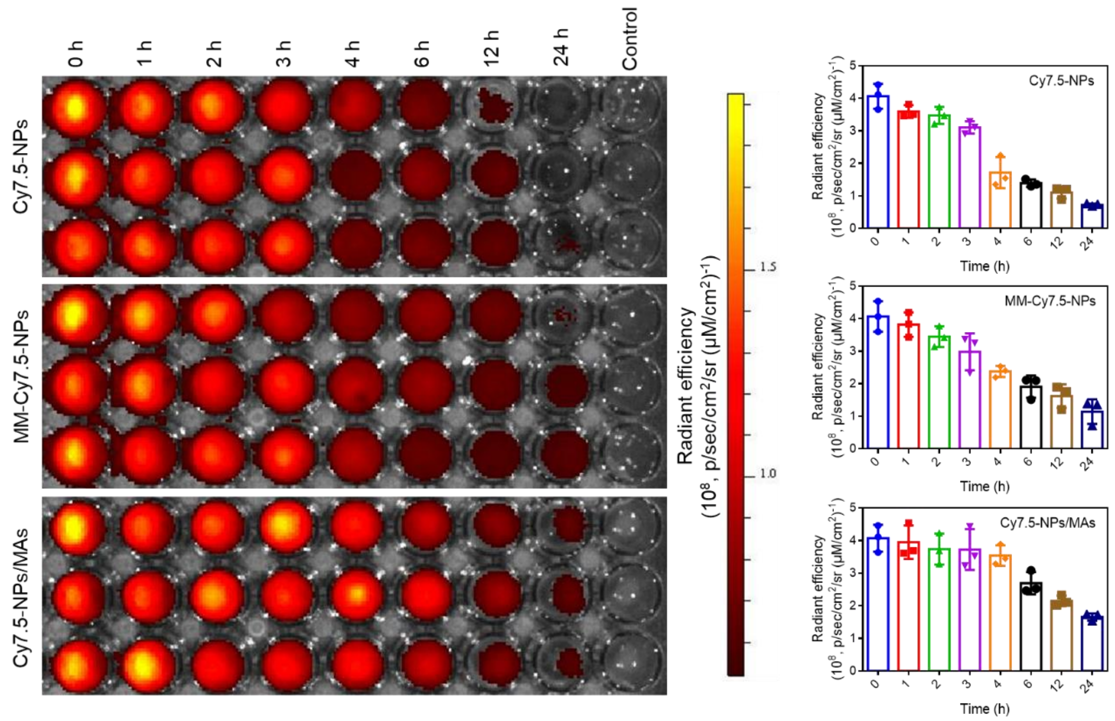

Supplementary Figure 11. *In vivo* pharmacokinetics of Cy7.5-NPs, MM-Cy7.5-NPs and Cy7.5-NPs/MAs in a mouse model. C57BL/6 mice were *i.v.* injected with Cy7.5-NPs, MM-Cy7.5-NPs and Cy7.5-NPs/MAs with the same dosage of Cy7.5 (2 mg kg^{-1}), and the blood was collected at predetermined time intervals. The fluorescence intensity was subsequently measured by *in vivo* imaging system (excitation at 780±20 nm, emission at 840±20 nm). All experiments were repeated for three times (*n* = 3) and data was presented as mean ± s.d. Source data are provided as a Source Data file.

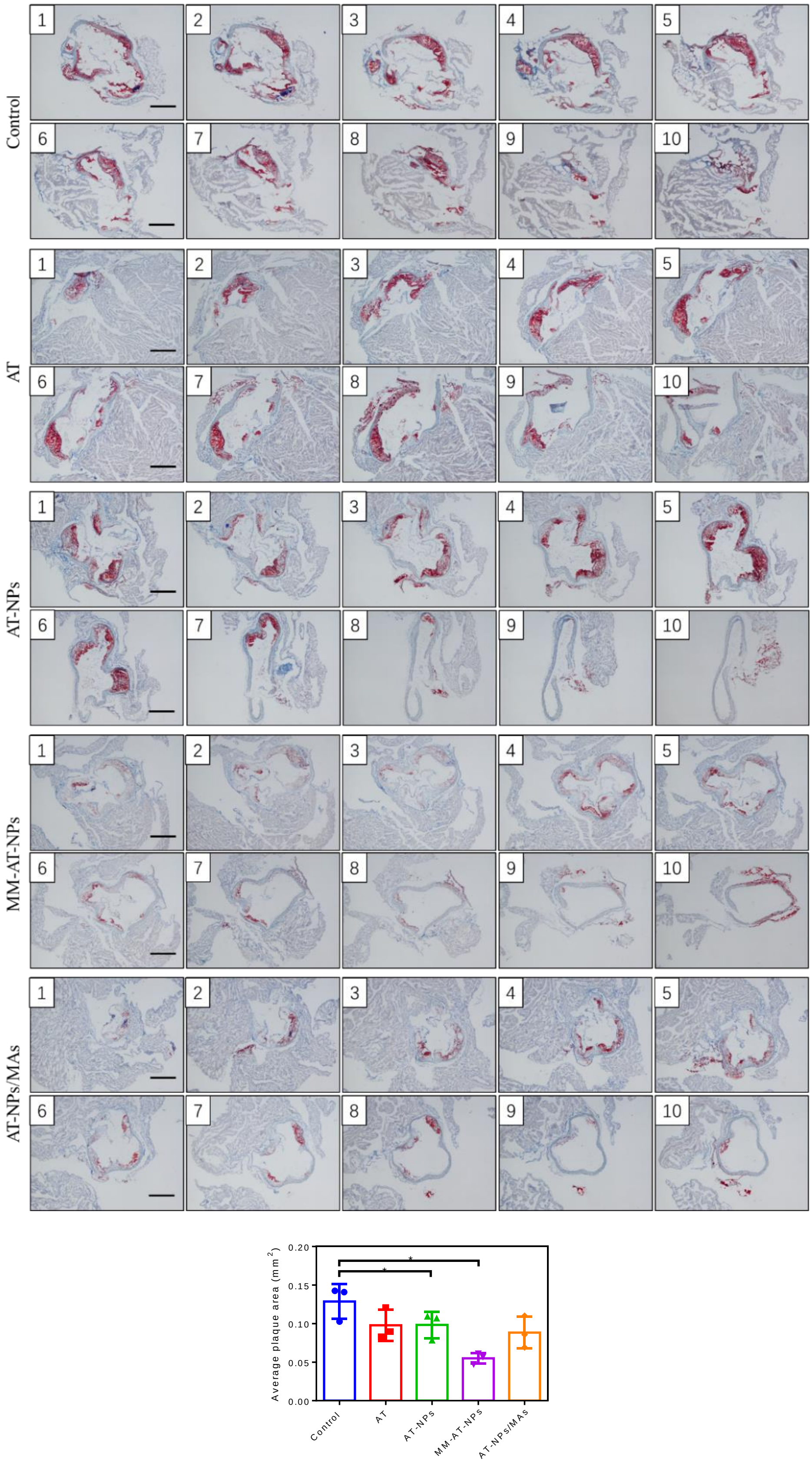

Supplementary Figure 12. ORO staining of aorta roots. Representative ORO stained aorta roots with sequential 10 cryosections at 100 μm intervals (top panel), and average plaque area of whole sections determined by Image Pro (bottom panel). Scale bar: 500 μm. All data were presented as mean ± s.d (n = 6). Statistical analysis was conducted using One-Way ANOVA. *P≤0.05. Source data are provided as a Source Data file.

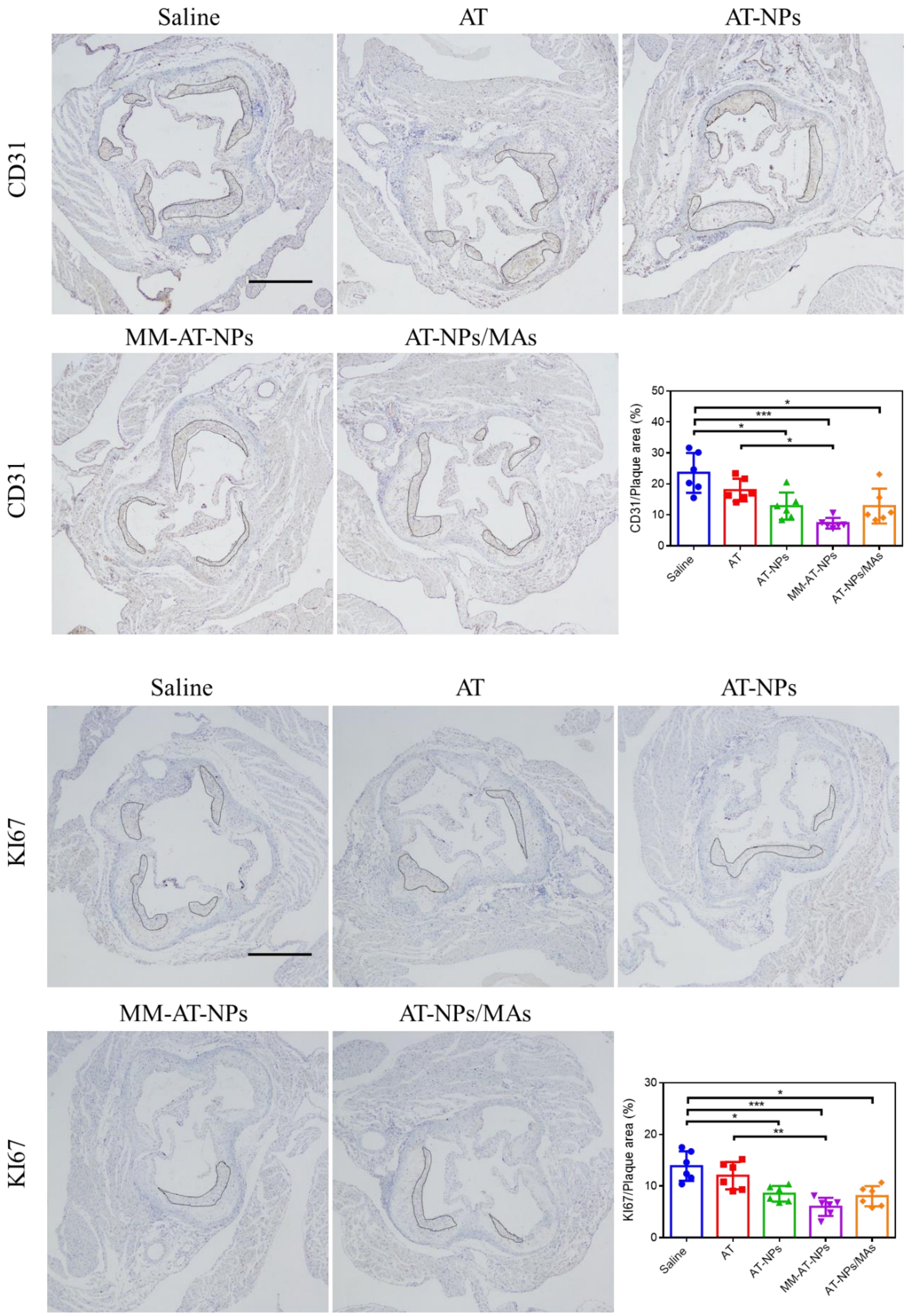

Supplementary Figure 13. Antibody staining analysis of aorta root. Representative photographs and quantitative analysis of aorta root sections stained by CD31 antibody (top panel) and KI67 antibody (bottom panel). Scale bar: 500 μm. All data were presented as mean ± s.d (n = 6). Statistical analysis was conducted using One-Way ANOVA. *P≤0.05, **P≤0.01, and ***P≤0.001. Source data are provided as a Source Data file.

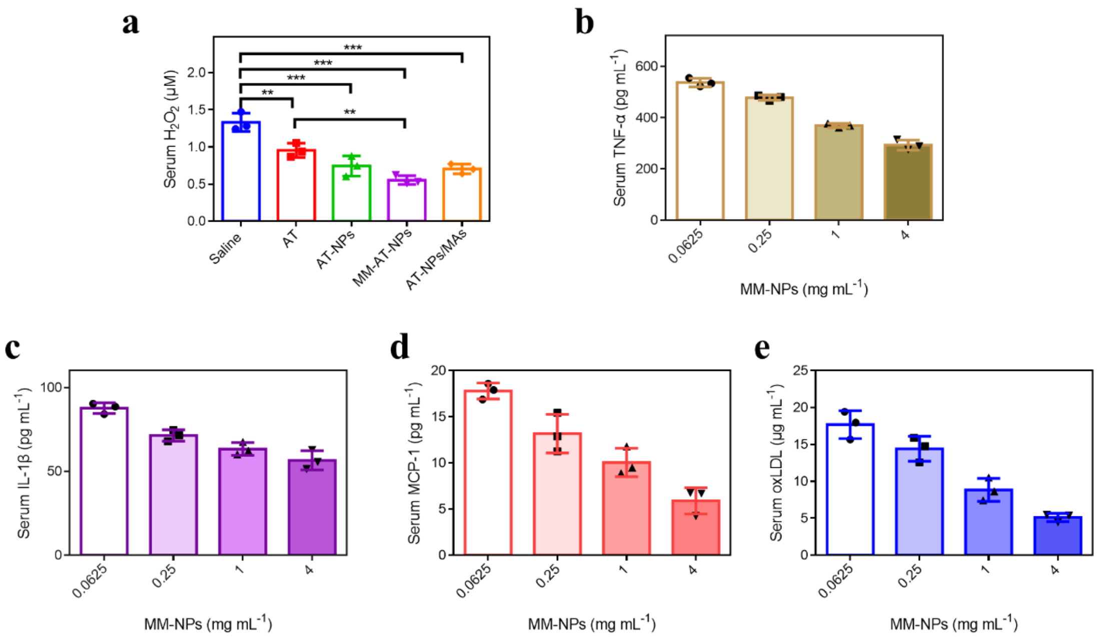

Supplementary Figure 14. Mechanism of anti-atherosclerosis by MM-AT-NPs. a, The level of H_2O_2 in the serum collected from atherosclerotic mouse after treatment with various formulations (AT, AT-NPs, MM-AT-NPs and AT-NPs/MAs) at dosage of 2 mg kg^{-1} AT per week. All data were presented as mean ± s.d (n = 3). Statistical analysis was conducted using One-Way ANOVA. $^{**}P\leq0.01$ and $^{***}P\leq0.001$. **b**,**c**,**d**,**e**, Binding affinity of MM-NPs with TNF-α, IL-1β, MCP-1 and oxPL-LDL in the serum collected from $ApoE^{-/-}$ mice after being fed with high-fat food for three months. All experiments were repeated for three times (n = 3) and data was presented as mean ± s.d. Source data are provided as a Source Data file.
