# Supplementary material for: Treatment of atherosclerosis by macrophage-biomimetic nanoparticles via targeted pharmacotherapy and sequestration of proinflammatory cytokines
Source: Nat Commun. 2020 May 26;11:2622. doi: 10.1038/s41467-020-16439-7 (PMC7251120; doi:10.1038/s41467-020-16439-7)
Supplement: Supplementary file 3 — Reporting Summary [file 41467_2020_16439_MOESM3_ESM.pdf]

nature research

Corresponding author(s): Ruibing Wang

Last updated by author(s): Mar 11, 2020

Reporting Summary

Nature Research wishes to improve the reproducibility of the work that we publish. This form provides structure for consistency and transparency in reporting. For further information on Nature Research policies, see Authors & Referees and the Editorial Policy Checklist.

Statistics

For all statistical analyses, confirm that the following items are present in the figure legend, table legend, main text, or Methods section.

n/a	Confirmed	
☐	☒	The exact sample size (*n*) for each experimental group/condition, given as a discrete number and unit of measurement
☐	☒	A statement on whether measurements were taken from distinct samples or whether the same sample was measured repeatedly
☐	☒	The statistical test(s) used AND whether they are one- or two-sided<br>*Only common tests should be described solely by name; describe more complex techniques in the Methods section.*
☐	☒	A description of all covariates tested
☐	☒	A description of any assumptions or corrections, such as tests of normality and adjustment for multiple comparisons
☐	☒	A full description of the statistical parameters including central tendency (e.g. means) or other basic estimates (e.g. regression coefficient) AND variation (e.g. standard deviation) or associated estimates of uncertainty (e.g. confidence intervals)
☐	☒	For null hypothesis testing, the test statistic (e.g. *F*, *t*, *r*) with confidence intervals, effect sizes, degrees of freedom and *P* value noted<br>*Give P values as exact values whenever suitable.*
☒	☐	For Bayesian analysis, information on the choice of priors and Markov chain Monte Carlo settings
☒	☐	For hierarchical and complex designs, identification of the appropriate level for tests and full reporting of outcomes
☒	☐	Estimates of effect sizes (e.g. Cohen's *d*, Pearson's *r*), indicating how they were calculated

Our web collection on statistics for biologists contains articles on many of the points above.

Software and code

Policy information about availability of computer code

Data collection	Flow cytometry data was collected by using BD Accuri C6 Software (version 1. 0. 264. 21). SoftMax Pro 5.4.1 was used for plate based absorbance data collection. IVIS (Lumina XR III) was used for in vivo fluorescence imaging and LAS X (version 3.5.2) was used for in vitro fluorescence imaging. Gatan DigitalMicrograph 3.9 was used for TEM. Zetasizer Software (version 7.11) was used for DLS/zeta.
Data analysis	Graphpad Prism 6 were used for statistical analysis and data plotting. FlowJo software (version 7.6.1) was used for flow cytometry analysis. Image-Pro Plus 6.0 was used to analyze fluorescent image.

For manuscripts utilizing custom algorithms or software that are central to the research but not yet described in published literature, software must be made available to editors/reviewers. We strongly encourage code deposition in a community repository (e.g. GitHub). See the Nature Research guidelines for submitting code & software for further information.

Data

Policy information about availability of data

All manuscripts must include a data availability statement. This statement should provide the following information, where applicable:

- Accession codes, unique identifiers, or web links for publicly available datasets
- A list of figures that have associated raw data
- A description of any restrictions on data availability

The source data underlying Figs. 1c-d, 1g–h, 1i, 2a, 2c-d, 2e, 2f, 3c, 3f, 4a, 4b-g, 4h, 4i, 5b, 5c-d and 5f and Supplementary Figs. 3a, 3b, 3c, 4a-4b, 4c, 4f, 4h, 6, 8b-d, 9a, 9b, 10, 11, 12, 13 and 14a-e are provided as a Source Data file. The data supporting all the plots within this paper are available from the corresponding authors upon request. A reporting summary is available as a Supplementary Information file.

Field-specific reporting

Please select the one below that is the best fit for your research. If you are not sure, read the appropriate sections before making your selection.

☒ Life sciences ☐ Behavioural & social sciences ☐ Ecological, evolutionary & environmental sciences

For a reference copy of the document with all sections, see nature.com/documents/nr-reporting-summary-flat.pdf

Life sciences study design

All studies must disclose on these points even when the disclosure is negative.

Sample size	The source of each cell line was stated in Methods and figure legends. Sample size were estimated to achieve about 90% power for detection of significant differences between groups based on means and standard deviations.
Data exclusions	No data were excluded from the analysis.
Replication	All the experiments were repeated for three times independently.
Randomization	The samples for in vivo and in vitro were randomly allocated into different experimental groups.
Blinding	The investigators were blinded to group allocation during experiments, data collection and analysis.

Reporting for specific materials, systems and methods

We require information from authors about some types of materials, experimental systems and methods used in many studies. Here, indicate whether each material, system or method listed is relevant to your study. If you are not sure if a list item applies to your research, read the appropriate section before selecting a response.

Materials & experimental systems

n/a	Involved in the study	
☐	☒	Antibodies
☐	☒	Eukaryotic cell lines
☒	☐	Palaeontology
☐	☒	Animals and other organisms
☒	☐	Human research participants
☒	☐	Clinical data

Methods

n/a	Involved in the study	
☒	☐	ChIP-seq
☐	☒	Flow cytometry
☒	☐	MRI-based neuroimaging

Antibodies

Antibodies used

The following antibodies were used for WB analysis.
CCR-2 Rabbit polyclonal antibody (catalog number: ABP53395, Abkine)
TNFR2 Rabbit polyclonal antibody (catalog number: ABP52623, Abkine)
CD36 Rabbit Polyclonal antibody (catalog number: 18836-1-AP, Proteintech)
GAPDH (14C10) Rabbit mAb (Biotinylated) antibody (catalog number: #5014, Cell Signaling Technology)
Horseradish peroxidase–conjugated anti-rabbit IgG antibody (catalog number: #7074, Cell Signaling Technology)
The following antibodies were used for immunohistochemical analysis.
CD31 Rabbit polyclonal antibody (catalog number: GB11063-3, Servicebio)
Ki67 Rabbit polyclonal antibody (catalog number: GB13030-2, Servicebio)
CD14 Rabbit polyclonal antibody (catalog number: GB11390-1, Servicebio)
MMP9 mouse polyclonal antibody (catalog number: GB12132, Servicebio)
α-SMA mouse polyclonal antibody (catalog number: GB13044, Servicebio)
CD68 mouse polyclonal antibody (catelog number:GB14043, Servicebio)
Goat anti-rabbit IgG antibody (catelog number: GB1213, Servicebio)
Goat anti-mouse IgG antibody (catelog number: GB1214, Servicebio)

Validation

The validation and application of antibodies were also mentioned in the manuscripts, in the method part
CCR-2 Rabbit polyclonal antibody (catalog number: ABP53395, Abkine), species: Mouse, Rat, validated by SDS-PAGE
TNFR2 Rabbit polyclonal antibody (catalog number: ABP52623, Abkine), species: Human, Mouse, Rat, validated by SDS-PAGE
CD36 Rabbit Polyclonal antibody (catalog number: 18836-1-AP, Proteintech), species: human, mouse, hamster, piglet, swine, validated by gene Knockout method
GAPDH (14C10) Rabbit mAb (Biotinylated) antibody (catalog number: #5014, Cell Signaling Technology) specity: H M R Mk B Pg, validated by siRNA knockout method, phosphatase or activator treatment, and then analyse between various cell lines and batches
CD31 Rabbit polyclonal antibody (catalog number: GB11063-3, Servicebio), species: Human, Mouse, validated by Biochips

Ki67 Rabbit polyclonal antibody (catalog number: GB13030-2, Servicebio), species: Human, Mouse, validated by Biochips
CD14 Rabbit polyclonal antibody (catalog number: GB11390-1, Servicebio), species: Human, Mouse, validated by Biochips
MMP9 mouse polyclonal antibody (catalog number: GB12132, Servicebio), species: Human, Mouse, validated by Biochips
α-SMA mouse polyclonal antibody (catalog number: GB13044, Servicebio), species: Human, Mouse, validated by Biochips
CD68 mouse polyclonal antibody (catelog number:GB14043, Servicebio), species: Human, Mouse, validated by Biochips

Eukaryotic cell lines

Policy information about cell lines

Cell line source(s)	Human umbilical vein endothelial cell HUVEC and mouse macrophage cell line RAW264.7 were purchased from ATCC (USA)
Authentication	These cell lines were authenticated by cell vitality test, isozyme detection, DNA fingerprinting, and mycoplasma detection.
Mycoplasma contamination	The cell lines were detected for mycoplasma contamination and no mycoplasma was found.
Commonly misidentified lines (See ICLAC register)	No commonly misidentified cell lines were used.

Animals and other organisms

Policy information about studies involving animals; ARRIVE guidelines recommended for reporting animal research

Laboratory animals	6-week-old female ApoE-/- mice and 6-week-old female C57BL/6 mice used in this study were maintained in a dedicated pathogen-free animal facility with free access to food and water in the Institute of Chinese Medical Sciences, University of Macau.
Wild animals	This study did not involve wild animals.
Field-collected samples	This study did not involve field-collected samples.
Ethics oversight	All animal procedures were approved by the Animal Ethics Committee, University of Macau.

Note that full information on the approval of the study protocol must also be provided in the manuscript.

Flow Cytometry

Plots

Confirm that:

☒ The axis labels state the marker and fluorochrome used (e.g. CD4-FITC).

☒ The axis scales are clearly visible. Include numbers along axes only for bottom left plot of group (a 'group' is an analysis of identical markers).

☒ All plots are contour plots with outliers or pseudocolor plots.

☒ A numerical value for number of cells or percentage (with statistics) is provided.

Methodology

Sample preparation	Cells are detached from flasks and immediately washed with PBS before Flow cytometry analysis.
Instrument	BD Acurri C6 cytometer
Software	BD Accuri C6 Software (version 1. 0. 264. 21) and FlowJo 7.6 were used for flow cytometry analysis.
Cell population abundance	No post-sort fractions were collected. Flow cytometry was used for quantitative analysis only.
Gating strategy	Initial cell populations were gated for a live population using FSC and SSC plot of cell only sample. The gate was set to remove cell debris and dead cells (small FSC and SSC).

☒ Tick this box to confirm that a figure exemplifying the gating strategy is provided in the Supplementary Information.
